# Variability of doublecortin-associated dendrite maturation in adult hippocampal neurogenesis is independent of the regulation of precursor cell proliferation

**DOI:** 10.1186/1471-2202-7-77

**Published:** 2006-11-15

**Authors:** Tobias Plümpe, Dan Ehninger, Barbara Steiner, Friederike Klempin, Sebastian Jessberger, Moritz Brandt, Benedikt Römer, Gerardo Ramirez Rodriguez, Golo Kronenberg, Gerd Kempermann

**Affiliations:** 1 Max Delbrück Center for Molecular Medicine (MDC) Berlin-Buch, Germany; 2 Volkswagenstiftung Research Group, Dept. of Experimental Neurology, Charité University Medicine, Berlin, Germany; 3 UCLA Medical Center, Departments of Neurobiology, Psychiatry and Psychology, 695 Charles Young Drive South, Los Angeles, CA 90095, USA; 4 The Salk Institute for Biological Studies, Laboratory of Genetics, 10010 North Torrey Pines Rd. La Jolla, CA 92037, USA; 5 Zentralinstitut für Seelische Gesundheit, J5, 68159 Mannheim, Germany

## Abstract

**Background:**

In the course of adult hippocampal neurogenesis most regulation takes place during the phase of doublecortin (DCX) expression, either as pro-proliferative effect on precursor cells or as survival-promoting effect on postmitotic cells. We here obtained quantitative data about the proliferative population and the dynamics of postmitotic dendrite development during the period of DCX expression. The question was, whether any indication could be obtained that the initiation of dendrite development is timely bound to the exit from the cell cycle. Alternatively, the temporal course of morphological maturation might be subject to additional regulatory events.

**Results:**

We found that (1) 20% of the DCX population were precursor cells in cell cycle, whereas more than 70% were postmitotic, (2) the time span until newborn cells had reached the most mature stage associated with DCX expression varied between 3 days and several weeks, (3) positive or negative regulation of precursor cell proliferation did not alter the pattern and dynamics of dendrite development. Dendrite maturation was largely independent of close contacts to astrocytes.

**Conclusion:**

These data imply that dendrite maturation of immature neurons is initiated at varying times after cell cycle exit, is variable in duration, and is controlled independently of the regulation of precursor cell proliferation. We conclude that in addition to the major regulatory events in cell proliferation and selective survival, additional micro-regulatory events influence the course of adult hippocampal neurogenesis.

## Background

Neuronal development in the adult hippocampus proceeds from the division of a precursor cell to a mature new granule cell [1,2]. A large surplus of neurons is generated, from which only a subset is recruited for survival and long-term integration into the network. This process is activity-dependently regulated. Once cells have been selected they have a lasting existence [3]. Adult hippocampal neurogenesis appears to originate from an astrocyte-like precursor cell (type-1 cell/vertical astrocyte) that gives rise to a transiently amplifying progenitor cell (type-2 cell) [4,5]. At this stage first indications of neuronal determination are found. The cells receive, for example, their first synaptic input, which is GABAergic [6,7]. The newly generated cells also begin to express doublecortin (DCX) [8-12].

DCX is a protein associated with neuronal differentiation that functionally has been linked to cell migration and possible other aspects of maturation, including synaptogenesis [13]. Mutations of the DCX gene cause migration disorders such as cortical laminar heterotopia and the doublecortex syndrome (hence the name) [14-17]. DCX expression is not limited to areas of neurogenesis but the exact function of DCX in these regions is not known yet. In the adult dentate gyrus, DCX is only expressed in cells contributing to adult neurogenesis.

In adult hippocampal neurogenesis, the phase of DCX expression coincides with the expression of the polysialylated form of the neural cell adhesion molecule (PSA-NCAM). DCX is transiently expressed during a period that extends from a proliferative progenitor cell stage (type-2b/3) to a postmitotic phase with long dendrites [5]. The phase of DCX expression is thus associated with neurite elongation and the DCX protein has been detected in the growth cones of dendrites and axons [11,18]. On average, this phase appears to last approximately 3 weeks [8,19]. DCX is absent from mature granule cells. All migration appears to occur during the period of DCX expression, but the majority of new cells remains within the inner third of the granule cell layer [3]. Because DCX is localized in the cytoplasm of immature neurons, their morphology becomes appreciable in immunohistochemical studies with antibodies against DCX [8-10,20]. Under naïve conditions, i.e. the absence of neurogenic stimuli, the dentate gyrus of a 2 month-old C57BL/6 mouse contains about 7000 DCX-expressing cells [21]. With increasing age, when adult neurogenesis decreases, the total number of DCX-positive cells is reduced to only about 120 at the age of 2 years. At two months of age, about 17% of all dividing cells in the subgranular zone (SGZ) of the dentate gyrus were DCX-positive [22]. The present study was done in 6 to 8 week-old mice, that is in young adulthood.

Regulation of adult hippocampal neurogenesis occurs primarily on two stages, both of which might be linked to DCX expression: expansion of the precursor cell pool and selective recruitment of new neurons [23]. We hypothesize that the proliferative regulation is relatively non-specific, whereas the survival effect is elicited by specific hippocampal activity, most notably learning. We have previously found that physiologic pro-proliferative stimuli such as voluntary physical activity primarily affects early progenitor cells (type-2 cells) [22]. Experimental seizures, in contrast, had a non-exclusive but particular effect on the proliferation of late progenitor cells (type-3 cells) [24]. However, judging from the steep decline in the number of surviving new cells with increasing time after exit from the cell cycle, the activity-dependent survival effect also takes place still during the phase of DCX expression and on a rather immature yet postmitotic stage.

Based on these observations we asked: what is the actual time course of the postmitotic maturation process, most notably with respect to dendrite development, and is it homogenous? Van Praag et al. have described that it takes approximately 7 weeks until by electrophysiological standards the newborn granule cells have become largely indistinguishable from their older neighbors and many studies in several laboratories currently try to link morphological and functional maturation [25,26]. Zhao and colleagues have studied green fluorescent protein (GFP)-labeled newborn neurons to describe the details of dendrite development [27]. Jones and colleagues studied patterns in dendrite maturation of granule cells early postnatally [28].

We wondered if the dynamics of dendrite development were dependent only on the time of the exit from the cell cycle or if any indication could be obtained that the dendritic maturation might be subject to additional regulation. In other words: would non-specific stimuli be acting on a precursor cell stage have consequences on later maturation as well? To address such mechanistic questions it would be necessary to describe the dynamics of early dendritic development during the DCX phase of adult neuronal development. Our aim was to better describe the course of neuronal development in the adult hippocampus encompassing the transition from the expansion phase to functional selection.

## Results

### DCX-expressing cells show a range of morphologies reflecting neuronal differentiation

We first aimed at describing early dendritic development and linking it to mitotic and post-mitotic stages of neuronal development in the adult hippocampus.

**Figure 1:**
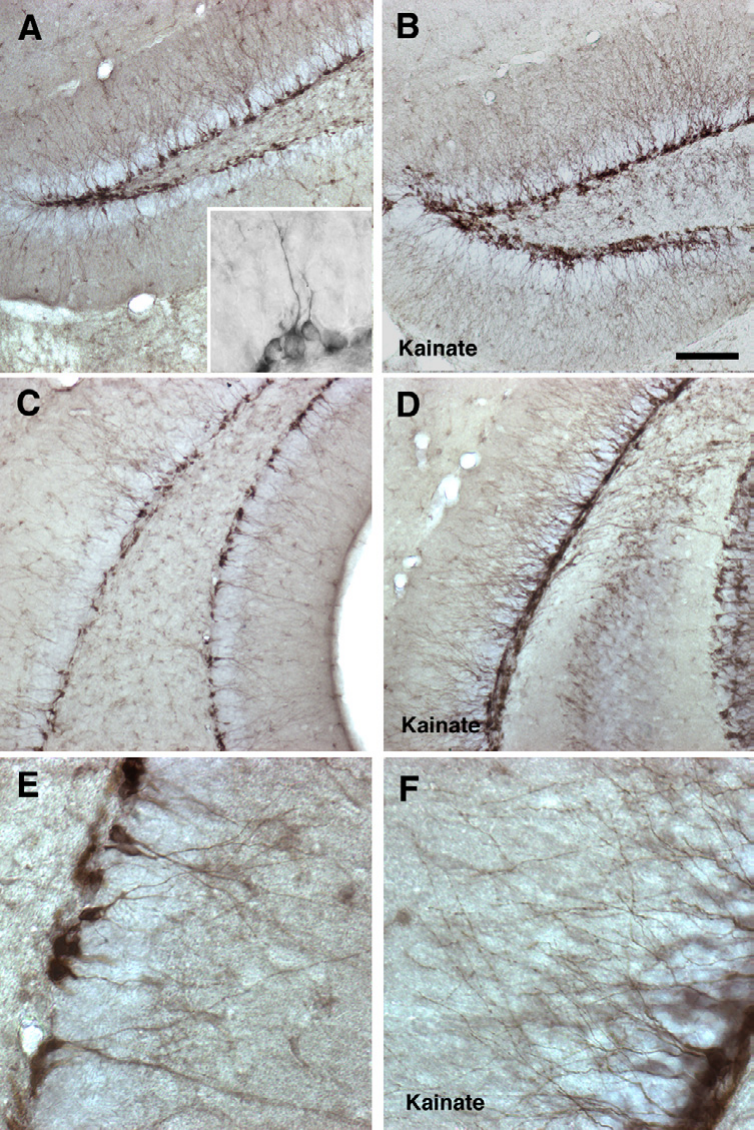
**DCX-expressing cells in the subgranular zone**. Column A, C, D shows normal conditions, column B, D, F shows conditions after induction of seizures by kainate application. **A and C**, DCX-positive cells in the subgranular zone (SGZ) with dendrites of various lengths reaching into the molecular layer. The nucleus appears usually devoid of immunoreaction. The granule cell layer is seen as a light band. Scale bar (in B for all panels), 150 μm. Insert, high power depiction of a cluster of DCX-positive cells of different morphology. Note, how the initial segment of the dendrite grows parallel to the SGZ. Scale bar, 45 μm. **B and D**, Kainate application increases the number of DCX-positive cells in the SGZ (here at 10 days after seizure induction). The SGZ widens and occasionally shows a bi-layered structure. Scale bar, 150 μm. **E and F**, Kainate injections increase the number of DCX-positive cells but do not seem to cause abnormal growth of the apical dendrites. Scale bar, 80 μm.

On tissue sections of the adult hippocampus overviews (Fig. 1A, C, E) showed the band of DCX-positive nuclei in the SGZ and a few DCX-positive cells in the granule cell layer proper. The dendritic trees are visible and reach far into the molecular layer. They were, as far as this can be judged from light-microscopic images, devoid of dendritic spines. This pattern is in accordance with the literature and our own previous reports [8-11,24,27]. In addition, a growth pattern of transient basal dendrites has been described for rats [18,29,30]. Basal dendrites were not readily discernible by DCX-immunohistochemistry in our mice. This is in accordance with previous data from our group [24] and studies based on a POMC-GFP reporter gene construct [31,32].

**Figure 2:**
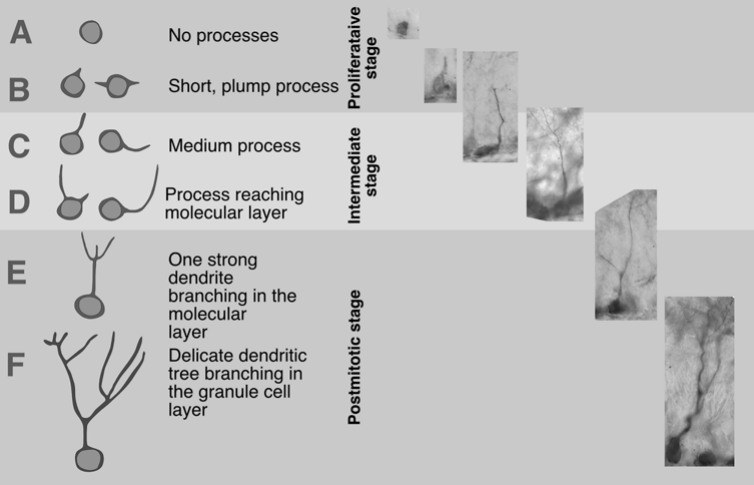
**Categorization of dendritic morphology**. We devised a schema of six categories of dendritic morphology in order to facilitate the semiquantitative description of the development of DCX-positive cells. The focus is on apical dendrites because basal dendrites were not clearly discernible under physiological conditions. In reality, the six categories, A through F, reflect a continuum. The right half of the figure shows light microscopic images of DCX expressing cells in the SGZ to exemplify the six categories of maturation.

**Figure 3:**
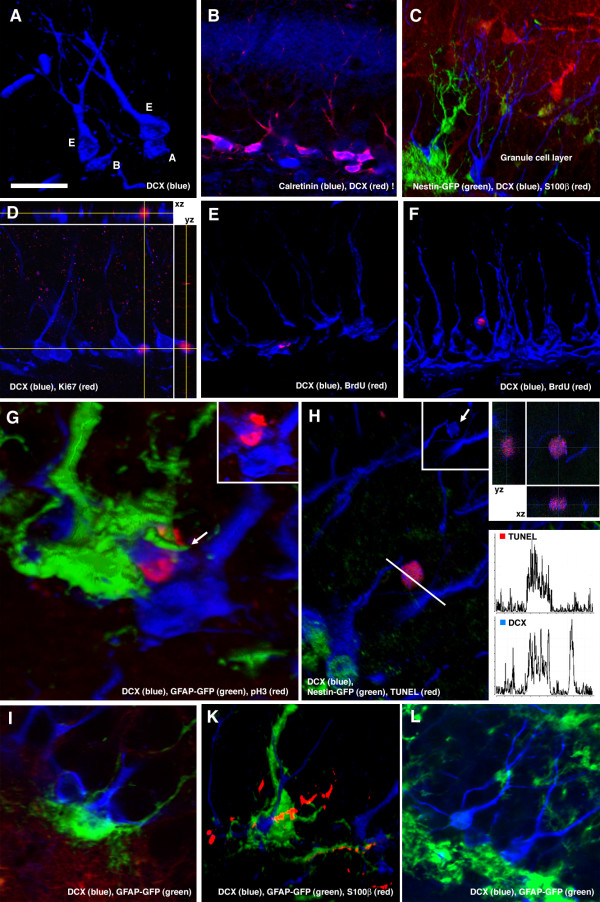
**Confocal microscopy of DCX-positive cells**. **A**, three-dimensional reconstruction of confocal microscopic z-series through sections stained for DCX expression. Two cells with no or short processes (A and B) are shown and two examples of cells in category E. Among all DCX-positive cells, category E is most abundant, accounting for more than 50% (see Fig. 4B) of the cells. Scale bar (in A for all panels), 30 μm. **B**, Co-localization of DCX and calretinin (CR) expression. CR expression identifies a postmitotic phase of granule cell development [8]. About 70% of all CR-positive cells are DCX-positive. The remaining DCX-positive CR-negative cells can be found in all categories except F (cf. Fig. 6B). Conversely, as depicted here, CR-positive cells can be found in all six categories. This also implies that (as suggested by the morphology) cells in categories A and B become postmitotic. The time span between exit from the cell cycle and the onset of CR expression varies. Single optical plane. Scale bar, 70 μm. **C**, many DCX-positive cells are in close contact to nestin-positive radial cells, here visualized in a nestin-GFP reporter gene mouse [19, 69]. These "vertical astrocytes" are considered the stem cells of the neurogenic region of the SGZ [1]. They are consistently S100β-negative [5]. 3-D reconstruction. Scale bar, 100 μm. **D**, Ki67 identifies a DCX-expressing cell with category A morphology as dividing. The panels to the right and on the top depict reconstructions from a confocal z-stack in xz and yz direction to confirm that the Ki67-positive nucleus belongs in fact to the DCX-positive cell. Scale bar, 70 μm. **E**, three-dimensional reconstruction of a BrdU-labeled cell with B morphology, 4 h after BrdU-injection. Scale bar, 70 μm. **F**, three-dimensional reconstruction of a BrdU-labeled cell with D morphology, 3 days after BrdU-injection. Scale bar, 70 μm.**G**, DCX-positive cells of all six morphological categories can be found in contact with astrocytes. In this example, during mitosis a GFAP-GFP-positive process surrounds the dividing DCX-expressing cell in the division plane. The separated sets of chromosomes were visualized with immunohistochemistry against phosphorylated histone H3 (red). Scale bar, 15 μm. **H**, apoptotic cells in the SGZ and granule cell layer were visualized with the TUNEL method (red). In the adult SGZ, TUNEL-positive cells are rare. Only 32 cells were found in 37 animals. Of these, a substantial number were DCX-positive (arrow in inset; DCX, blue), implying that cell death occurs indeed on the level of DCX expression. Co-localization was further demonstrated by placing virtual slices in *yz *and *xz *direction through the cell in question (top right) and by measuring the intensity of the fluorescent signals along a line placed across the cell (bottom right). The curves for the TUNEL signal (top, red) and DCX (bottom, blue) are at the same position. Single optical plane. Scale bar, 50 μm. **I**, the processes of GFAP-positive cells that engulf DCX-positive cells sometimes form a basket "cradling" the DCX-expressing cell. 3-D reconstruction. Scale bar, 15 μm. **K**, the vertical astrocytes with their close spatial relationship to DCX-positive cells are negative for S100β (red), whereas processes of horizontal astrocytes (arrow) are positive for GFAP-GFP and DCX. 3-D reconstruction. Scale bar, 50 μm. **L**, not all DCX-positive cells have close contact to astrocytes as visualized with the GFAP-GFP reporter gene mouse. 3-D reconstruction. Scale bar, 50 μm.

Morphological examination of DCX expressing cells in the SGZ showed a range of morphologies. We categorized DCX-expressing cells into 6 categories according to the presence and the shape of apical dendrites (Fig. 1A inset and 1C) and their presumed sequential order (Fig. 2). Category A and B were cells with no or very short processes, C and D cells with processes of intermediate length and immature morphology, and E and F cells with a more mature appearance. Process length was less than one nucleus-wide (< 10 μm) in B. In category C the process was longer than in B, reached into the granule cell layer but did not touch the molecular layer. In category D, the process touched the molecular layer. In groups B, C and D we often found that the process did not leave the cell perpendicular to the SGZ but had at least an initial segment that went parallel or oblique to the SGZ before curving into the depth of the granule cell layer (Fig. 3A and 3D). In category E one thick dendrite reached into the molecular layer and showed a comparatively sparse branching in the molecular layer. In category F, the dendritic tree showed delicate branching and few major branches either near the soma or within the granule cell layer. In reality, a continuum between these categories exists.

DCX is thus expressed during the initial steps of neuronal differentiation, beginning with un-polarized cells with processes parallel to the granule cell layer and ending with a highly polarized cell that extends processes into the molecular layer and shows dendritic branching. All DCX-positive cells in the SGZ and granule cell layer could be categorized according to these criteria. This is in accordance with the idea that during adult hippocampal neurogenesis DCX expression is restricted to the neuronal lineage. Also, morphologically distinguishable interneurons such as basket cells were DCX-negative.

**Figure 4:**
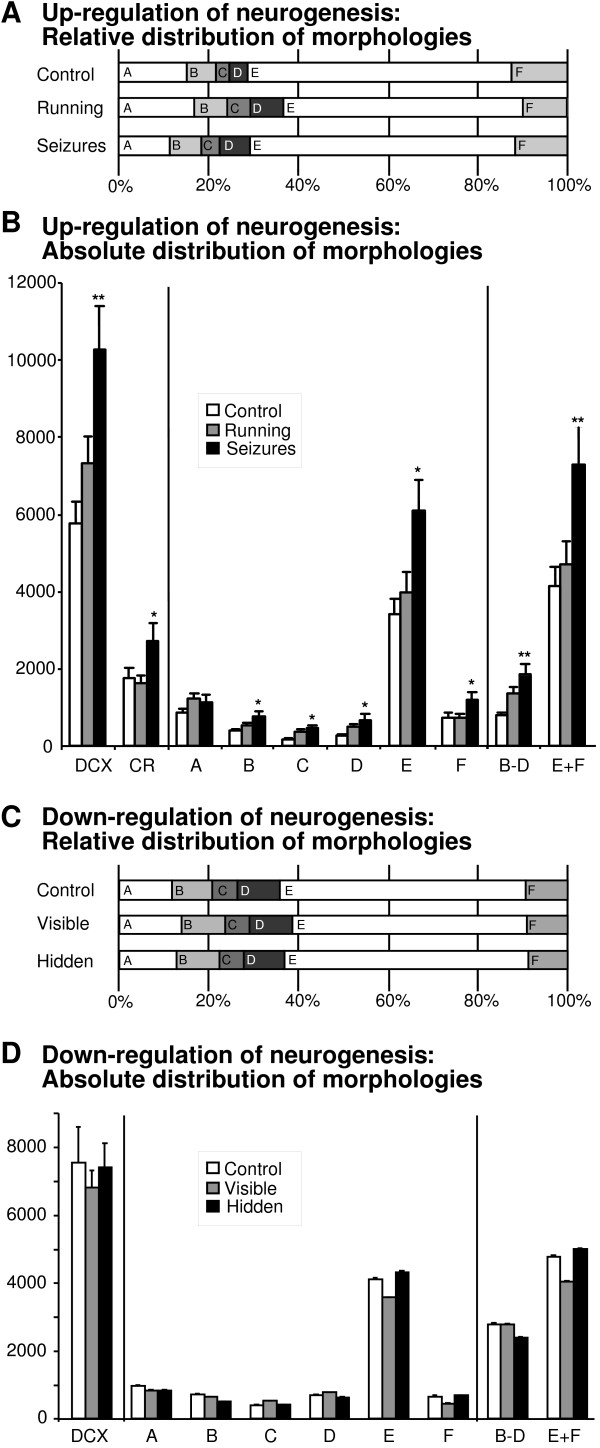
**Distribution of dendritic morphologies among DCX-positive cells**. **A**, among all DCX-positive cells of the SGZ, 22% are in categories A and B, the actively dividing stages (cf. Fig. 2). If categories C and D are included in this consideration the fraction of potentially proliferative DCX-positive cells is about one third (cf. Fig. 5). More than 50% of the DCX cells are in category E. If adult hippocampal neurogenesis is increased by voluntary wheel running or kainic acid-induced seizures, at the peak of cell proliferation the distribution of DCX-positive cells is not changed compared to control conditions. **B **depicts the absolute numbers. Across the categories there is an increase in cell numbers. **C**, stress associated with swimming in the Morris water maze was found downregulate adult neurogenesis and affected dividing precursor cells [44]. Analysis of dendritic morphology of DCX-positive cells from that study revealed again no change in the distribution of dendritic morphologies. **D **shows the absolute values.

We quantified the distribution of DCX-positive cells to these phenotypes and found that more than 65% of the DCX-positive cells belonged to categories E and F (Fig. 4A, values for "Control"). Less than 10% were found in the intermediate categories C and D; groups A and B accounted for roughly 20%. This indicates that at any given time point, DCX identifies a majority of cells with a relatively mature phenotype.

**Figure 5:**
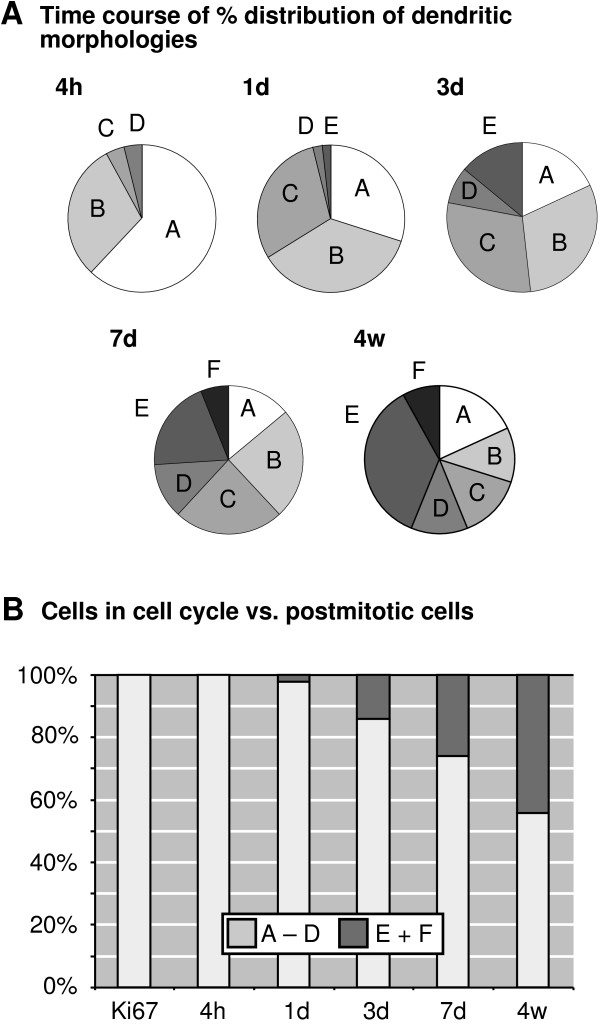
**Distribution of dendritic morphologies at different time points after cell division**. **A**, with increasing time after labeling DCX-positive cells with BrdU, the cells progress through the 6 categories of dendritic morphology (cf. Fig. 2). At 4 weeks after BrdU, 45% of the labeled cells have reached the most mature stages E and F. Note that the absolute number of BrdU-positive cells has decreased by this time due to an activity-dependent selection process [2, 8] and that apparently, cells down-regulate DCX soon after reaching category F. Consequently, the relatively large proportion of DCX-positive cells in the potentially proliferative stages A – D (**B**) corresponds to only a low absolute number at late time points after BrdU.

The proliferative activity of DCX expressing cells was examined with two independent immunohistochemical methods. We studied either the incorporation of permanent S-phase marker bromodeoxyuridine (BrdU) 4 hours after the injection of BrdU or the expression of cell cycle-associated protein mki67 with antibody Ki67 [33]. BrdU is incorporated into the DNA only during S-phase. Because of the length of the remaining cell cycle [34], at 4 hours after the injection the cells have not yet exited from the cell cycle and BrdU thus allows an estimate of cells that are currently dividing (i.e. are in S-phase or briefly thereafter). The protein recognized by antibody Ki67 (mki67) is endogenously expressed during late G1, S, G2, and M phases of the cell cycle but not in early G1 and G0 [33]. Both approaches revealed that proliferating DCX-positive cells (i.e. DCX-positive cells that were at least in S-phase) tended to be found in the less mature categories (Fig. 5A and 5B), but there were many examples of cells that had extended short dendrites and were incorporating BrdU or expressing mki67. Figure 3F shows an example of a BrdU-labeled cell with long dendrite. Such cells were comparatively rare and we can only speculate about the significance of this observation. The general conclusion from our investigation is not influenced by this rather surprising finding: Categories with smaller or no dendrites are those associated with cell cycle markers.

There were no dividing cells in categories E and F, which consequently reflect the postmitotic stages of DCX-expressing cells. We know that the postmitotic stage is also associated with the additional expression of NeuN and CR; CR-positive cells overall showed a morphology similar to DCX-expressing cells in categories E and F (compare also [8]). Overall, 71% of the CR-positive cells in the granule cell layer were also DCX-positive, 29% were DCX-negative.

Finally, we wanted to confirm that the phase of DCX expression would indeed also be associated with apoptotic cell death. Every 6^th ^section (at 40 μm) from 18 mice were processed for TUNEL (TdT-mediated X-dUTP nick end labeling), a feasible way to detect apoptosis. One series was co-stained for DCX-expression, one for CR-expression. TUNEL-positive cells were extremely rare in the hippocampal region. In both series together, only 37 TUNEL-positive cells were detected. These were located either within the SGZ (5 out of 37) or in the granule cell layer proper (32 out of 37). In the DCX-series, 5 out of 17 TUNEL-positive cells showed a DCX immunoreaction (Fig. 3H). In the CR-series, 2 out of 20 TUNEL-positive cells showed a CR-immunoreaction. No clear statement can be made about the cells apparently lacking the DCX- or CR-immunoreaction, because with increasing nuclear condensation the other phenotypic characteristics of the cells are lost. Also, our data do not imply that other eliminated cells would have to be DCX- or CR-negative.

### During the developmental phase of DCX-expression newborn cells are closely associated with astrocytes

Seki and Arai were the first to propose a tight association of developing new neurons and radial glia elements [35]. Seri et al. reported a close spatial relationship between DCX-positive cells and astrocytes of the SGZ [1]. Shapiro and colleagues have further examined this association and found that radial glia-like astrocytes would "cradle" newborn cells in the course of their development [36]. This partially engulfing relationship is different from the more confined contacts that essentially all neurons have with astrocytes. In both latter studies, the analysis was based on immunohistochemistry with antibodies against glial fibrillary acidic protein (GFAP) and electron microscopy. Seri and colleagues used PSA-NCAM expression to identify the intermediate stage of development [1].

We asked how the close association between maturing neurons and astrocyte-like cells would relate to the stages of maturation. To visualize GFAP-positive cells on a light microscopical level in their full morphology, we made use of a reporter gene mouse, which expresses GFP under the GFAP-promoter [37]. We found DCX-positive cells in various spatial relationships to GFAP-GFP-positive cells (Fig. 3G, I, K, and 3L).

DCX-positive cells either did not show any contact to GFAP-positive cells (6.8%), were touched by an astrocytic process (15.8%), or were surrounded by GFAP-positive processes like a basket (77.3%; Figs. 3G, I, K, or 3L). This "cradling" only in rarer cases appeared as complete as in Fig. 3I. Rather, the cells showed variable degrees of laminar contacts to GFAP-positive cells. Seri et al. have suggested that cradling might shield the new cells from the hilus; the basket-like structure would be open towards the granule cell layer. We could not confirm that this orientation was mandatory. Our data suggest a larger variability in the spatial relationship between astrocytes and DCX-positive cells. We could confirm, however, that most of the cradling astrocytes had a radial glia-like morphology. They were consistently negative for astrocytic marker S100β. This is consistent with our previous observation that the astrocyte-like precursor cells of the SGZ are GFAP-positive but S100β-negative [5,19]. Occasionally, a close proximity between GFAP/S100β-doublepositive astrocytes and DCX-positive cells was found but this rare event was confined to the narrow band of the SGZ proper and did not involve basket-like cradling. In any case, the enclosure of the DCX-positive cells by astrocytes often did not so much resemble a basket, but more a loop-like capture. Figure 3G shows an example, in which such a loop passes around a DCX-positive cell, which is in the M-phase of cell division, visualized by pH3-immunohistochemistry. The GFAP-positive process surrounds the DCX-positive cell exactly in the cleavage plane.

**Figure 6:**
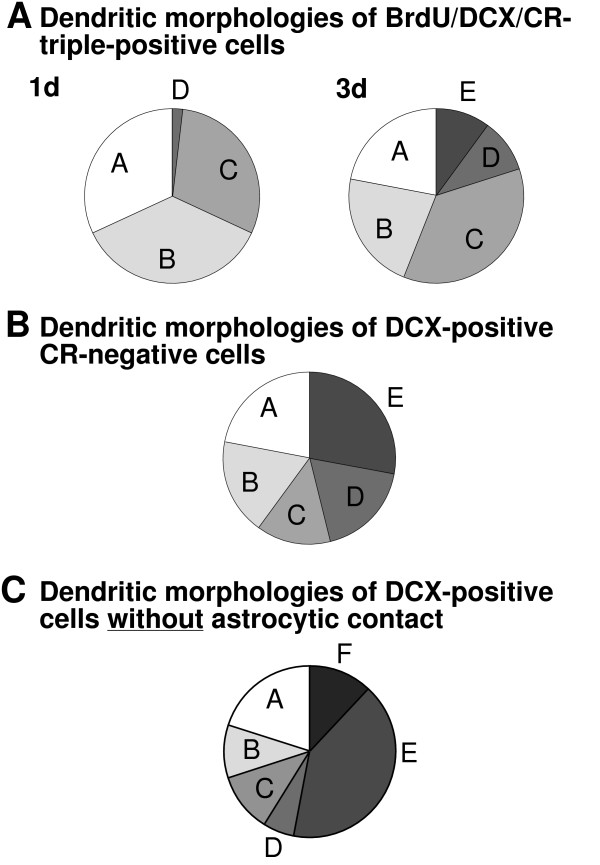
**Distribution of dendritic morphologies in other subgroups of cells**. **A**, BrdU/DCX/CR triple immunohistochemistry identifies cells that have just undergone cell division but have terminally exited from the cell cycle. Consequently, at 1 day after BrdU, these cells are found in categories A – D, two thirds in categories A and B. Two days later, about 10% have progressed to more advanced stage E. **B**, CR as a postmitotic marker in immature granule cells has the limitation that the onset of CR expression varies after exit from the cell cycle (cf. Fig. 3B). As a result, CR-negative DCX-positive cells can be found as late as stage E. However, they were never found in category F. **C**, in all categories cells without contact to astrocytes were found, but more than half of these cells were in the advanced categories E and F. If put into relation to the distribution of proliferative cells among DCX-positive cells and the absolute numbers (Fig. 4) this implies that during the early period of DCX-expression most cells have contacts with astrocytes but tend to lose this contact with increasing maturation.

We did not find a clear preferential association of the most immature categories with the astrocytes although more cells with astrocytic contact were found in categories A – D, and the cells seemed to lose their contacts with increasing maturation (Fig. 6C).

### The duration of DCX expression varies in the course of neuronal development

To assess the progression through the developmental stages, we used a time course study, in which BrdU was injected once and the brains were examined at different time points after the injection. The overall pattern was consistent with development in the sense of a progression from less mature to more mature appearing stages. At early time points after BrdU, DCX-positive cells were mainly found in categories A through C (Fig. 5); at 4 weeks after BrdU, the majority of cells had reached categories E and F. However, at late time points after BrdU (7 days and 4 weeks) a considerable number of DCX-positive cells was found in categories A through D, indicating either asymmetric divisions on these stages or strongly varying dynamics of development. As early as 1 day after BrdU the first cells were seen in postmitotic category E (but not F). This is consistent with previous observations that new neurons can show very rapid signs of postmitotic maturation after the last cell division [8,38] and the presence of BrdU/DCX/CR-triple-positive cells at 1 day after BrdU (Fig. 6A). Consequently, the total duration of the phase of DCX expression appears to vary. Even at 4 weeks after BrdU the number of cells in the most mature category F was low compared to the situation at 7 days after BrdU. Presumably, at stage F the new cells are all CR-positive and begin to down-regulate DCX [8].

At 1 and 3 days after BrdU, we examined the distribution of BrdU/DCX/CR-triple-positive cells into the morphological classes A through F (Fig. 6A). We found a shift towards more mature stages between 1 and 3 days. The absence of any BrdU/DCX/CR-triple-positive cells in category F at 3 days after BrdU told us that it takes more than 3 days after exit from the cell cycle to reach this level of maturity. But more importantly, we found BrdU/DCX/CR-triple-positive cells in the most immature stages. This implies that postmitotic cells are found in categories A, B, and C, which can otherwise (if CR negative) also be associated with proliferative activity. Cells might leave the cell cycle, while DCX-positive and express CR at varying time-points (here represented by the different categories) thereafter.

### Dynamic of neuronal maturation is not influenced by neurogenic stimuli

The observation of variable dynamics in maturation prompted us to ask whether these differences might reflect differential regulation. We thus studied the distribution of morphologies between DCX-positive cells in two experimental paradigms with known inducing effects on adult hippocampal neurogenesis and clear effects on proliferation of the precursor cells: voluntary wheel running [22,39] and kainic acid-induced seizures [24,40,41].

As described previously, in the case of wheel running we did not find a significant increase in the number of DCX-positive cells at this time-point but an increase in cell proliferation [21]. DCX-positive cells showed the same relative and (consequently absolute) distribution to the categories of dendritic maturation, indicating that no independent effect of voluntary exercise on this aspect of development existed (Fig. 4).

Induction of seizures by the systemic application of kainic acid strongly increased adult neurogenesis. One week after seizures, at the peak of the proliferative response (BrdU-positive cells: 4623.6 ± 658.7 vs. 764.0 ± 29.9 in controls; *p *< 0.0001), we determined the total number of DCX-expressing cells and found almost a doubling (Fig. 4B). Figure 1B, D, F show DCX immunohistochemistry after kainic acid induced seizures. As described previously the SGZ is widened in these animals and more DCX-positive cells are found within the granule cell layer [24]. In contrast to the report by Scharfman and colleagues from rats, we did not find large numbers of ectopic cells in the hilus [42].

When the DCX-positive cells were categorized into the classes A to F, we found that the distribution did not differ from controls. Although the absolute numbers in each category increased (significantly except for classes A and F), the distribution in the different categories was not influenced. We interpret this result as indicating that at one week after the neurogenic stimulus, progression of DCX-positive cells through the developmental stages highlighted by DCX expression was constant. The number of cells had increased but not their (variable) rate of further development. This might imply that the variability in the duration of DCX expression is not affected by the microenvironmental condition prevalent after experimental seizures. Whereas one study showed an acute proliferative response of GFAP-positive radial cells after experimental seizures [43], one of our own recent studies found that at the peak of proliferation after kainic acid-induced seizures the increased proliferative activity can be primarily attributed to DCX-expressing type-3 cells, many of which then show an ectopic position in the granule cell layer [24] (see also Fig. 1D). The present data add to this finding by suggesting that in seizures other aspects of neuronal maturation can remain relatively undisturbed as well. This is consistent with our observation that the excessively generated new neurons under seizures have survival times of at least months [24].

### Down-regulation of neurogenesis similarly does not affect dendritic maturation

We have recently shown that unlike in rats, training the Morris water maze led to a reduction in adult hippocampal neurogenesis in mice, presumably in response to the stress associated with swimming [44], although this explanation remains speculative at present. Stress is an often described negative regulator of adult hippocampal neurogenesis in both acute and chronic settings [45-48], but the exact mechanisms remain still unknown and some published studies have shown contradicting results [48,49]. In our study we had found an interesting difference between training the hidden and the cued version of the task [44]. Both versions of the test caused a decrease in proliferation of type-3 progenitor cells and CR-positive cells. The number of proliferative early progenitor cells, type-2 cells, of which only a subset is DCX-positive, was reduced only in the cued version, where the larger effect on neurogenesis was found. In the tissue still available from that experiment we now assessed the total number of DCX-expressing cells in the dentate gyrus and found no change. Microscopically, DCX expression in the dentate gyrus appeared indistinguishable from the distribution shown in Fig. 1A. Consequently, the differential regulation that was seen on the level of the proliferative DCX-positive cells was not reflected in the total population of DCX-expressing cells. When we analyzed the distribution of these cells on the different stages of dendritic development we found that like in the case of the up-regulation in response to synaptic activation, no change in the developmental pattern was seen. The distribution was very similar in mice trained on the hidden platform, on the visible platform and in controls (Fig. 3C, D). From this we conclude that the manipulation did not directly affect dendritic maturation in DCX-positive cells.

## Discussion

In the course of adult hippocampal neurogenesis, the newly generated neuronal cells pass through a stage that is characterized by transient DCX expression. In the present study we have added a number of important insights to the knowledge about this crucial phase, which links the precursor cell stage with a postmitotic immature neuronal stage. We show that the phase of increasing dendritic maturation can have variable length but that this pattern, despite its variability, is not influenced by either positive or negative neurogenic stimuli. We propose that these results indicate that although newborn neurons follow a rather fixed pattern of dendritic development and maturation (as captured by the categories A – F), initiation of this development is not only dependent on proliferation per se and exit from the cell cycle, and that regulation of neurogenesis does not overtly include aspects of dendritic maturation.

Whereas about 22% of the DCX positive cells were in cell cycle, 68% were showing a co-localization with the strictly postmitotic marker calretinin. This implies that at the given time-point, the remaining 10% of DCX-positive cells are in the progression from a progenitor cell to an early postmitotic stage. The most mature cells (based on dendritic morphology), were always CR-positive. But at one day after BrdU, some CR-positive cells without processes were found in the SGZ. About one third of the DCX-positive cells represent progenitor cells (type-2b and type-3 cells), whereas the remainder are immature postmitotic neurons. This ratio might not be fixed and may be subject to regulation or age-dependent shifts. Within the group of postmitotic cells the time point of CR expression after exit from the cell cycle also differed.

We used DCX as a marker here. DCX protein binds to elements of the cytoskeleton, including intermediate filament nestin, which is expressed in precursor cells. In general, DCX expression has been primarily brought into connection with neuronal migration, although other aspects of pathfinding including a role in the growth cones of neurites and in synapse formation have been suggested [14]. Because both processes involve cytoskeletal dynamics and DCX binds to the microtubular apparatus it is tempting to speculate that this association is causal. Our finding that although dendrite development follows a predictable pattern it is still variable in onset and duration, suggests that additional levels of regulation exist that become effective after DCX has started to be expressed. Neurogenic stimuli that act on precursor cells, such as those studied here, do not seem to belong to these regulators. We propose that regulation of dendritic maturation is influenced by those stimuli that rather affect the selective survival of newborn cells.

A future study will address this hypothesis. However, the experiment is not as straightforward as it might seem because activity-dependent survival is dependent on a critical postmitotic period. So far, no studies in mice have been able to unambiguously demonstrate *selective *survival, e.g. in response to a learning stimulus [39,44]. Also in rats, where positive results exist, the data are not yet fully conclusive [50-52].

The largest population of proliferative progenitor cells in the SGZ is DCX-negative. Pro-proliferative stimuli affect DCX-positive and DCX-negative cells [22]. Activity-dependent regulation of cell survival, in contrast, primarily affects cells that have left the cell cycle but still might be DCX-positive. From our previous [2] and the present data and consistent with other reports [53,54] we hypothesize that the phase of DCX and CR co-expression is the phase during which the newborn cells are primarily selected for long-term survival. Postmitotic cells within the neuronal lineage (e.g. expressing TUC4, associated with axon elongation, or the polysialylated form of the neural cell adhesion molecule, PSA-NCAM) have been shown to have a reduced threshold of LTP induction and thus are presumably particularly plastic [53-55].

We found that the newborn cells leave the close contact to astrocytes, which presumably represents the precursor cell niche, at varying times in the course of development, but separation from the niche is not predictive of the degree of (dendritic) maturation. This might indicate that once the stem cells have produced their differentiating progeny, other factors but the engulfing astrocytic processes become necessary. We and others hypothesize that these factors depend on synaptic input.

First synaptic input reaches the newly generated cells of the dentate gyrus on a nestin-positive progenitor cell level and this first input is GABAergic [6]. Ambrogini and colleagues have characterized the stages of development electrophysiologically and found GABAergic input as well [56]. They did not test for DCX expression in their study. Because nestin-GFP and DCX show an overlap in expression (type-2b cells) both studies should have identified the identical cell type. GABA, which acts excitatory at these early stages of development promotes further maturation of new granule cells [7,57].

Many current hypotheses on the activity-dependent regulation of adult hippocampal neurogenesis include a Hebbian mechanism, in which newly generated cells are recruited into the network based on the successful establishment of connectivity early after becoming postmitotic [51,58-60]. This hypothesis is supported by the finding that the number of surviving cells declines rapidly after the cells have turned postmitotic. We add to this idea of a selective induction of survival early after exit from the cell cycle by demonstrating that a number of the apoptotic cells in the SGZ were DCX- or CR-positive. Due to the very low number of TUNEL-positive cells in our sample, no quantitative statement is possible. We hypothesize from our data that dendritic development might in fact depend on this initial immature synaptic integration.

Our data indicate that the selection and elimination process might take place, when the newly generated cells already have acquired comparatively complex apical dendrites. Because this phase is also associated with TUC4 expression (not shown here) and TUC4 is involved in axon elongation, the selected cells also should have extended their axon to CA3. Nevertheless, it is not yet known, whether the synaptic input that mediates the survival-promoting effect is direct (e.g. from the perforant path), or indirect (e.g. via recurrent axon collaterals and GABAergic interneurons). In total, synaptic and functional maturation of new neurons takes several weeks and extends beyond the period of DCX and CR expression [25,61,62].

Synaptic activity is likely to promote cell survival in a similar manner than described for synaptic plasticity. For example, NMDA receptors are likely to be involved in mediating this effect, because blockade of NMDA receptors increased adult neurogenesis [63-66]. Massive synaptic (and extra-synaptic) stimulation of kainic acid and other excitatory receptors causes a strong and lasting increase in cell proliferation [40,41]. We have shown that at the peak of proliferative activity this increase is largely due to a division of DCX-expressing cells [24]. In the present study we found a corresponding increase in the total number of DCX-positive cells as well as an increase in the number of DCX-positive cells in the proliferative stages (A – D). Surprisingly, however, we also found similar increases in the remaining categories (E and F) and an overall distribution of dendrite morphologies that was not different from control conditions (Fig. 4).

This might suggest that the overall dynamics of apical dendrite development were not altered by the seizures. This is not to say that other parameters related to dendritic development not investigated here would have to remain unaffected as well. The morphology, extent and persistence of basal dendrites, for example, is strongly affected by experimental seizures [18,67].

DCX is thought to be primarily involved in neuronal migration [15], either by stabilizing and destabilizing protofilaments or by modulating dynein action [68]. Migration of new neurons in the SGZ is limited, and we have found previously that the immature cells find their final position early in development [3]. The extent and duration of DCX expression during adult hippocampal neurogenesis thus seems surprising. However, certain cytoskeletal rearrangements in the course of neurite extension might also rely on DCX function, although this remains to be shown. Alternatively, the variable duration of DCX expression as seen in our study might also reflect a varying migrational state, even if the actual migration is minimal. The migrational condition might in turn be associated with other developmentally relevant events. We found that in the initial phase of dendrite elongation, the immature neurite often paralleled the SGZ (or was oblique to it), whereas in the mature cells the stem of the dendritic tree is perpendicular to the SGZ. This might indicate that the cell bodies could follow the lead of the outgrowing dendrites and relocate to an appropriate position perpendicular to the stem. Migration in the SGZ might thus actually consist of a relocation of the cell body along the pioneering dendrite.

As a final note, our data indicate that assessing the number of DCX-positive cells in the dentate gyrus alone as a measure of "adult neurogenesis" might give a misleading impression, because the phase of DCX expression is not only associated with possible cell death (thus reducing quantitative precision) but also encompasses a wide range of qualitative changes.

## Conclusion

In this report we described that approximately 20% of the DCX expressing population in the adult hippocampus belong to the actively dividing precursor cells, whereas more than 70% of the DCX cells were found to be postmitotic. We found the period of time span until the newborn neurons had reached the most mature stage of dendritic maturation that is still associated with DCX expression varied in length between 3 days and several weeks. Intriguingly, both positive and negative regulators of precursor cell proliferation did not change the pattern and the dynamics of DCX-associated dendrite development. We interpret our findings as indicating that dendrite maturation of adult-generated hippocampal neurons begins at variable time-points after exit from the cell cycle and is variable in duration. Consequently, we hypothesize that the neuronal maturation phase is controlled largely independently from the regulation of precursor cell proliferation and cell cycle exit. This finding has interesting implications for our understanding of how adult hippocampal neurogenesis is regulated in that neuronal maturation does not follow an automated temporal program initiated with fate choice and cell cycle exit but is subject to additional micro-regulatory influences.

## Methods

### Animals

The experiments were largely done on material from a previously published study [8]. A set of 6 week-old 24 female C57BL/6 mice (19–24 g; Charles River) were divided into 6 groups and perfused 4 hours (N = 5), 1 day (N = 5), 3 days (N = 5), 1 week (N = 5), 2.5 weeks (N = 4) and 4 weeks (N = 5) after a single intraperitoneal BrdU-injection (5-Bromo-2-deoxyuridine, 50 mg/kg BrdU in sterile 0,9% NaCl; Sigma).

Additional 18 female C57BL/6 mice (same age as above) were divided into 3 groups: seizure (N = 6), running (N = 6) and controls (N = 6). Seizure animals received a single intraperitoneal application of 30 mg/kg kainic acid (KA, Sigma) in 0.1 M phosphate buffered saline (PBS) on the day before BrdU-injections (Day 0), and only those displaying continuous convulsive seizure activity were used in these experiments. The "Runner" group was housed with 2–3 animals per cage that was equipped with a running wheel. During the first 7 days of the experiment all animals received one daily injection of BrdU. Tissue from this experiment will be used in an unrelated study. Data on dendritic morphology, etc., were exclusively generated for the present study.

To analyze the spatial relationship between DCX-positive cells and astrocytes we used 3 female transgenic mice expressing enhanced green fluorescent protein EGFP under the promoter for glial fibrillary acidic protein (GFAP). The animals were kindly provided by Helmut Kettenmann, Berlin [37] and were 7 weeks of age. For the detection of apoptosis a total of 37 female C57BL/6J mice (Charles River, Sulzfeld, Germany), 8 weeks of age, were used.

For the water maze experiment, 21 female Nestin-GFP reporter mice [69] (C57BL/6, 10 weeks of age at the beginning of the experiment) were used and randomly assigned to one of the following experimental groups: Morris water maze hidden version, Morris water maze cued version, standard laboratory conditions. On the 3 days before the experiment each animal received single injection of BrdU. Animals were subjected to water maze training at days 5 to 8 of the experiment (days 5 to 8 after the last BrdU injection). We followed the protocol devised by Wolfer and Lipp [70]. Six trials of training each maximally lasting for 2 minutes were given each day. Tissue from that experiment that has been published elsewhere [44] was analyzed for the present study. Again, the data on dendritic morphology, etc., were exclusively generated for the present study.

### Tissue preparation

The mice were killed with an overdose of ketamine and perfused transcardially with 4% paraformaldehyde in 0.1 M phosphate buffer (pH 7.4). The brains were removed from the skull, were kept in the fixative for 24 h and then transferred into 30% sucrose, where they remained until equilibrium. Forty μm coronal sections were cut from a dry ice-cooled copper block on a sliding microtome (SM 200R, Leica). The sections were stored at -20°C in cryoprotectant containing 25% ethylene glycol and 25% glycerin in 0.05 M phosphate buffer. Sections were stained with free floating immunohistochemistry and pretreated for BrdU-detection by incubation in 2 N HCl for 30 min at 37°C followed by washing in 0.1 M borate buffer (pH 8.5) for 10 min.

Brain tissue for the TUNEL assay (see below) was rapidly frozen in liquid nitrogen and cut on a cryostat at 15 μm. Sections were mounted onto Superfrost coated glass slides, air-dried, post-fixed with 4% paraformaldehyde (20 min) and thoroughly washed in phosphate buffered saline (PBS).

### Immunohistochemistry

To determine the number of BrdU-labeled and DCX-expressing cells we used the peroxidase method (ABC system, Vectastain, Vector Laboratories) with biotinylated donkey anti-rat and anti-rabbit antibodies (1:500; Dianova) and nickel-intensified diaminobenzidine as chromogen. Immunofluorescent triple labeling was done as described earlier [3].

The primary antibodies used in our study were monoclonal rat anti-BrdU (1:500; Biozol), monoclonal mouse anti-NeuN (1:100; Chemicon), monoclonal mouse anti-Calretinin (1:250; Swant), rabbit anti-S100β (1:250; Swant), goat anti-Doublecortin (1:200; Santa Cruz Biotech), polyclonal rabbit anti-Ki67 (1:500; Novocastra); polyclonal rabbit anti-GFP (1:400; Abcam); monoclonal mouse anti-phospho histone H3 (1:50; Cell Signalling Technology).

As fluorescent secondary antibodies we used anti-rat or anti-mouse Rhodamine-X, anti-mouse or anti-rabbit FITC, anti-mouse, anti-rabbit or anti-goat Cy5 (all 1:250; Jackson Laboratories, distributor: Dianova). Fluorescent sections were mounted in polyvinyl alcohol with diazabicyclo-octane (DABCO) as anti-fading agent.

### Western blot

We confirmed the specificity of our antibody against doublecortin, on which the results of the present study are based, using a western blot of homogenate from mouse hippocampus (not shown). The antibody showed the expected band below 50 kD. This reaction was completely blocked with the DCX peptide.

Mouse hippocampi were lysed with RIPA buffer (150 mM NaCl, 10% glycerol, 0.5 mM EDTA, 0.5% triton X-100, 1 mM phenylmethylsulphonyl fluoride, 25 μg/ml leupeptin, 25 μg/ml aprotinin and 1 mM sodium orthovanadate in 50 mM Tris-HCl, pH 7.6) and homogenized with an ultrasonic homogenizer (setting 40 Hz) for 60 seconds and cellular debris was removed by 15 minutes centrifugation at 14 000 rpm. Total protein content was quantified using a Bio-Rad protein reagent (Bio-Rad Laboratories GmbH. München). Aliquots of 40 μg of protein were separated by electrophoresis on 12% SDS-polyacrylamide gels by the Laemmli method [71] and transferred to PVDF paper. Membranes were blocked with 5% skim milk in 0.5% Tween 20-TBS (TTBS) and incubated with the goat polyclonal anti-doublecortin antibody diluted 1:200 overnight. The blots were washed 3 times with TTBS and incubated for 1 h in a 1:3000 dilution of peroxidase conjugated donkey anti-goat. The protein was visualized with the enhanced chemiluminescence detection system (ECL, Pierce GmbH. Bonn). The anti-doublecortin antibody specificity was tested using the blocking peptide as negative control (sc-8066P).

### Quantification of BrdU and DCX-labeled cells

From all animals series of every 6^th ^section (240 μm apart) were stained for BrdU- and for DCX with the peroxidase method. Positive cells were counted using a 40 × objective (Leica) throughout the rostro-caudal extent of the granule cell layer. As described previously, the optical dissector method was modified in that cells appearing sharp in the uppermost focal plane were not counted [3]. Resulting numbers were multiplied by 6 to obtain the estimated total number of BrdU- and DCX-positive cells per granule cell layer.

To categorize DCX-positive cells according to the 6 morphological grouping criteria described in this study, 100 DCX-labeled cells were analyzed per animal.

### Analysis of newly generated cell phenotypes

One-in-twelve series of sections from animals of each group were triple-labeled for BrdU, GFP and DCX or double-labeled for BrdU and DCX. Fluorescent signals were detected using a spectral confocal microscope (Leica TCS SP2). All analyses were performed in sequential scanning mode to rule out cross-bleeding between channels. Double-labeling was confirmed by three-dimensional reconstructions of z-series covering the entire nucleus (or cell) in question. Three-dimensional reconstructions were performed with Volocity 2.0 software (Improvision).

From each animal and for every combination of phenotypes 50 BrdU-positive cells within the granule cell layer were analyzed at the confocal microscope for co-expression of the different markers. Relative numbers were related to the absolute counts of BrdU-positive cells per granule cell layer to yield the absolute numbers of BrdU-marked cells per phenotype. Images were processed with Adobe Photoshop 7.0, and only general contrast enhancements and color level adjustments were carried out.

### TUNEL assay

The TUNEL (terminal desoxy transferase-mediated dUTP nick end labeling) method visualizes apoptotic cells by detecting free 3' ends of DNA strand breaks [72]. We used the ApopTag Fluorescein kit (Quantum Appligene). Brain sections were delipidated in ethanol/acetic acid (2:1) at -20°C. Afterwards the sections were washed and treated with the equilibrium buffer provided with the kit. The TUNEL reaction with digoxigenin-11-dUTP followed during 1 h at 37°C. Afterwards the reaction was stopped with the provided Stop/Wash-buffer for 10 minutes. The sections were thoroughly washed in PBS and incubated with either antibodies against DCX or CR (see above). The secondary antibodies were applied together with FITC-conjugated anti-digoxigenin antibodies. After another round of washes in PBS and destilled water, sections were air-dried and coverslipped as described above.

### Statistical analyses

All numerical analyses were performed using Statview 5.0.1. For all comparisons ANOVA was performed followed by Fisher's post hoc test, if appropriate. Differences were considered statistically significant at p < 0.05. The post hoc analyses were additionally run with Students-Newman-Keuls test, which did not yield differing results (not shown).

## Authors' contributions

TP, SJ and GK designed the study; TP, DE, BS, FK, MB, BR, GKr, and GR, designed and carried out the individual experiments and acquired the data; TP, SJ, BS, GR, GKr, and GK analyzed the findings in the light of the present hypothesis; TP and GK wrote the manuscript with support from the other co-authors. All authors read and approved the final manuscript.
